# Feasibility of an Emergency Department-Based Two-Step ADHD Screening Pathway in Primary School-Age Children Presenting with Home Accidents: A Prospective Two-Center Case-Control Study

**DOI:** 10.3390/children13091240

**Published:** 2026-09-13

**Authors:** Hilal Nur Beyoglu, Omer Basay, Reşad Beyoğlu

**Affiliations:** 1 Department of Child and Adolescent Psychiatry, Bandirma Training and Research Hospital, Balikesir 10200, Turkey; 2 Department of Child and Adolescent Psychiatry, Faculty of Medicine, Pamukkale University, Denizli 20070, Turkey; 3 Department of Emergency Medicine, Faculty of Medicine, Bandirma Onyedi Eylul University, Balikesir 10250, Turkey

**Keywords:** attention-deficit/hyperactivity disorder, ADHD, home accident, child injury, emergency department, feasibility, case-control study, DDDRS

## Abstract

**Background/Objectives:** Emergency department (ED) visits for pediatric home accidents may provide an opportunity to identify children who warrant evaluation for attention-deficit/hyperactivity disorder (ADHD). This study primarily evaluated the feasibility of a two-step ED-based ADHD screening and Child and Adolescent Psychiatry referral pathway. A secondary exploratory objective was to compare the frequency of confirmed ADHD between children presenting with home accidents and non-injured ED controls. **Methods:** This prospective, two-center case-control study was conducted in two emergency departments in Denizli, Turkey, between October 2022 and June 2023. Children aged 5–11 years were enrolled as home-accident cases or non-injured controls. Parents or guardians completed the Turkish-validated Turgay DSM-IV Disruptive Behavior Disorders Rating Scale (DDDRS). Screen-positive children and those with prior documented ADHD were referred for DSM-5-based diagnostic confirmation or record verification by Child and Adolescent Psychiatry specialists blinded to group allocation. Feasibility outcomes included completion of ED screening, referral threshold positivity, and attendance at specialist assessment. **Results:** The study included 139 home-accident cases and 139 controls. Among 267 children eligible for ED screening, all completed the DDDRS. Forty-nine children (18.4%) met the referral threshold, and 40 of 49 (81.6%) attended the Child and Adolescent Psychiatry assessment. Overall, ADHD was confirmed in 21 cases (15.1%) and 9 controls (6.5%) (unadjusted odds ratio [OR], 2.57; 95% confidence interval [CI], 1.13–5.83; *p* = 0.020). In an exploratory multivariable logistic regression model adjusted for sex, age, and school grade, confirmed ADHD remained associated with home-accident case status (adjusted OR, 2.45; 95% CI, 1.06–5.65; *p* = 0.035). However, a sensitivity analysis restricted to children without prior treated ADHD showed a non-significant difference in newly diagnosed ADHD (12/130 vs. 7/137; OR, 1.89; 95% CI, 0.72–4.96; *p* = 0.190). **Conclusions:** A two-step ED screening and specialist referral pathway for ADHD after pediatric home-accident presentation was operationally feasible in this two-center sample. The higher overall frequency of confirmed ADHD among home-accident cases should be interpreted as a secondary exploratory finding because it was partly influenced by imbalance in pre-existing ADHD and was not confirmed in the newly diagnosed-only analysis. Larger studies with center-level data, broader confounder measurement, and longitudinal follow-up are needed.

## 1. Introduction

Attention-deficit/hyperactivity disorder (ADHD) is the most prevalent neurodevelopmental disorder of childhood, characterized by developmentally inappropriate and persistent inattention, hyperactivity, and impulsivity that impairs functioning across multiple settings [1,2]. Epidemiological meta-analyses report a worldwide pooled prevalence of approximately 5–7% among school-age children [3], with estimates reaching 19.5% in Turkish cohorts [4]. Males are more frequently affected than females, with clinical sex ratios of approximately 2–3:1 [4,5].

Current diagnostic standards require that ADHD symptoms be developmentally inappropriate, persistent, associated with impairment, and present in more than one setting. Screening questionnaires can support case identification, but they do not substitute for diagnostic confirmation by a trained clinician using DSM-based criteria and information from more than one context whenever feasible [1,6].

Home accidents are a major source of pediatric morbidity and mortality worldwide. Recent pediatric emergency and injury-prevention literature emphasizes that unintentional household injuries remain common, preventable, and closely linked to age, supervision, domestic hazards, and social context [7,8,9]. In Turkey, home accidents represent an important component of pediatric injury-related ED attendances [10,11]. Preschool and primary school-age children are disproportionately affected because they spend substantial time in domestic environments and engage in exploratory, physically active behavior.

The biological and behavioral mechanisms linking ADHD to injury risk are plausible and clinically relevant. Core deficits in executive function, particularly response inhibition and working memory, reduce a child’s capacity to anticipate hazards, suppress impulsive motor actions, and maintain situational awareness [12,13]. A recent systematic review concluded that ADHD is associated with accident and injury risk across the lifespan, while the magnitude and injury type vary by age and context [14]. Studies involving specific pediatric injury subtypes have reported higher ADHD frequencies among injured children than among non-injured controls [15,16].

However, two issues remain underexplored. First, little is known about whether a brief ED-administered ADHD symptom screen followed by specialist referral is feasible after a pediatric home-accident presentation. Second, the specific relationship between confirmed ADHD and home accidents, as distinct from all injuries, has not been adequately characterized. The present study therefore aimed primarily to evaluate the feasibility of a two-step ED ADHD screening and Child and Adolescent Psychiatry referral pathway. A secondary exploratory aim was to compare confirmed ADHD frequency between primary school-age home-accident cases and non-injured ED controls, while describing sex and age patterns without inferential emphasis.

## 2. Materials and Methods

### 2.1. Study Design and Setting

This was a prospective, two-center, analytic case-control study conducted between 20 October 2022 and 20 June 2023 at the EDs of Pamukkale University Faculty of Medicine Hospital (annual census: approximately 140,000 visits) and Denizli Servergazi State Hospital (annual census: approximately 200,000 visits), Denizli, Turkey. Ethics approval was granted by the Pamukkale University Non-Interventional Clinical Research Ethics Committee (Decision No. 15; 18 October 2022) and the Denizli Provincial Directorate of Health. Written informed consent was obtained from all parents or guardians; verbal assent was obtained from children aged 7 years and older.

### 2.2. Participants and Matching

The case group comprised children aged 5–11 years enrolled in primary school (grades 1–4) presenting due to a home accident, defined as an unintentional injury occurring inside or immediately adjacent to the domestic residence, including balconies, staircases, and garden areas, while excluding traffic, school, sport, and outdoor public-space accidents. The control group comprised children of the same age and school grade who presented to the same EDs for non-traumatic, non-accident reasons during the same period. Controls were enrolled using frequency matching to achieve comparable sex distributions; formal one-to-one matching was not performed.

Enrollment was prospective during the study period. Detailed screening logs for all potential control candidates, recruitment hours, day-by-day recruitment windows, and center-specific enrollment counts were not retained in the anonymized analysis file. This limitation is acknowledged below because it constrains assessment of selection bias and center effects.

### 2.3. Inclusion and Exclusion Criteria

Inclusion criteria were age 5–11 years, enrollment in primary school, presentation during the enrollment period, and written parental or guardian consent. Five-year-old children were included only if they were already enrolled in primary school; they were not recruited from preschool settings. Exclusion criteria were age outside the primary school range; any unintentional injury in the preceding seven days (applied to controls only, to avoid a carry-over effect); comorbid psychiatric disorder other than ADHD, neurological disorder, or metabolic disorder; documented visual impairment; suspicion of neurological disease; developmental delay; or withdrawal at any stage. Acute infections, including upper respiratory tract infection and gastroenteritis, were not exclusion criteria because excluding them would have introduced selection bias in the control group, as these conditions represent common reasons for pediatric ED attendance.

Developmental delay was assessed through parental history and available medical records at enrollment; formal psychometric testing of intellectual functioning was not performed as part of this ED-based study. The exclusion of comorbid psychiatric disorders was intended to reduce diagnostic heterogeneity for this first feasibility-oriented analysis, but it produced a selected ADHD sample and limits generalizability to the broader clinical ADHD population, in whom psychiatric and neurodevelopmental comorbidity is common [17,18].

### 2.4. Sample Size

The original sample size calculation assumed an ADHD prevalence of approximately 13% in injured children versus 6% in controls, with two-sided alpha = 0.05 and power = 80%. During revision, this calculation was re-evaluated in response to reviewer comments. Under standard two-independent proportion assumptions, detecting a difference between 13% and 6% with 80% power would require approximately 267 participants per group. Therefore, the enrolled sample of 139 participants per group provides only approximately 53% power for this etiologic case-control comparison. Consequently, the present study is now framed primarily as an exploratory feasibility evaluation of the ED screening/referral pathway rather than a definitively powered study of ADHD as an independent risk factor for home accidents.

### 2.5. ADHD Screening and Diagnostic Confirmation

All eligible participants without prior treated ADHD were assessed in the ED using the Turkish-validated Turgay DSM-IV Disruptive Behavior Disorders Rating Scale (DDDRS), adapted for Turkish children by Ercan et al. [19]. The DDDRS contains 18 ADHD items, including nine inattention items and nine hyperactivity/impulsivity items, each scored from 0 to 3. The DDDRS was used as a referral screen, not as a diagnostic instrument.

A pragmatic referral threshold of five or more items rated “quite a lot” (score 2) or “very much” (score 3) in either domain was selected a priori to reduce false-negative referrals near the DSM symptom boundary. This threshold is one item below the six-symptom DSM threshold and was used to increase clinical sensitivity in the ED context. However, this specific referral threshold has not been independently validated as a stand-alone diagnostic cut-off, and its sensitivity and specificity in this setting cannot be estimated because screen-negative children did not undergo specialist diagnostic evaluation.

Screen-positive children and children with prior documented ADHD were referred to the Child and Adolescent Psychiatry (CAP) outpatient clinic. Diagnostic confirmation was performed by CAP specialists who were blinded to group allocation. The assessment was a specialist clinical DSM-5 evaluation rather than a fully structured research interview. It included a clinical interview with the parent or guardian and the child, a review of ED referral information, and assessment of symptom persistence, impairment, and cross-situational functioning based on parent-reported home and school histories and available clinical documentation. Teacher rating forms were not collected during the acute ED-based pathway. Each child was evaluated by a CAP specialist; formal inter-rater reliability was not calculated.

Children with a prior documented ADHD diagnosis who were currently receiving pharmacological treatment were classified as ADHD-confirmed after verification of available clinical records and current treatment history, without re-screening, because active treatment could attenuate parent-reported symptoms and produce artificially low DDDRS scores. The previous diagnosis was accepted if it was documented in clinical records as an ADHD diagnosis established by a physician or child/adolescent psychiatry service. Medication type, dose, treatment duration, and adherence were not systematically abstracted and therefore could not be analyzed as modifiers of injury risk. Children with a previous ADHD diagnosis but no active pharmacological treatment were not automatically classified as confirmed ADHD; they followed the ED screening and CAP referral procedure according to their DDDRS result.

The use of a DSM-IV-based screening questionnaire followed by DSM-5-based diagnostic confirmation is procedurally consistent because the core ADHD symptom criteria are substantially comparable across both versions for this age group. Nevertheless, the lack of teacher forms and structured diagnostic interviews is an important limitation of the diagnostic procedure [6,20].

### 2.6. Analytic Strategy and Missing Data

The primary feasibility outcomes were DDDRS completion among children eligible for ED screening, DDDRS referral-threshold positivity, attendance at CAP evaluation among referred children, and completion of diagnostic confirmation or record verification. The case-control comparison of confirmed ADHD frequency was treated as a secondary exploratory outcome.

The primary analysis used all 278 enrolled participants as the denominator. Nine children referred to CAP who did not attend their appointment (case: n = 4; control: n = 5) were treated as ADHD-unconfirmed in the primary analysis to preserve the intent-to-screen denominator; however, because absence of diagnostic assessment is not equivalent to a true negative result, a complete-case sensitivity analysis excluding these children was also performed. A separate sensitivity analysis was restricted to newly diagnosed ADHD among children without prior treated ADHD to examine whether the case-control finding persisted after removal of the pre-existing ADHD imbalance.

### 2.7. Statistical Analysis

Data were analyzed using SPSS version 22.0 (IBM Corp., Armonk, NY, USA). Categorical variables were compared using Pearson’s chi-square test or Fisher’s exact test, with Fisher’s exact test preferred when any expected cell count was below 5. Continuous variables were compared using the independent-samples *t*-test or Mann–Whitney U test, as appropriate. The unadjusted OR and 95% CI for ADHD diagnosis were calculated from 2 × 2 contingency tables. After individual-level age and school-grade data were provided during revision, an exploratory multivariable logistic regression model was performed with home-accident case status as the dependent variable and confirmed ADHD as the principal independent variable, adjusted for sex, age, and school grade. Study center, parental ADHD, socioeconomic position, household crowding, supervision level, previous injury, medication exposure, and other comorbidities could not be included because these variables were not available as individual-level covariates in the analysis dataset.

Pre-specified subgroup analyses involving sex and age group were conducted for descriptive purposes only; given the sample size and multiplicity of comparisons, subgroup results are reported as frequencies without inferential emphasis. Statistical significance was set at two-sided *p* < 0.05.

## 3. Results

### 3.1. Participant Flow and Feasibility Outcomes

**Figure 1:**
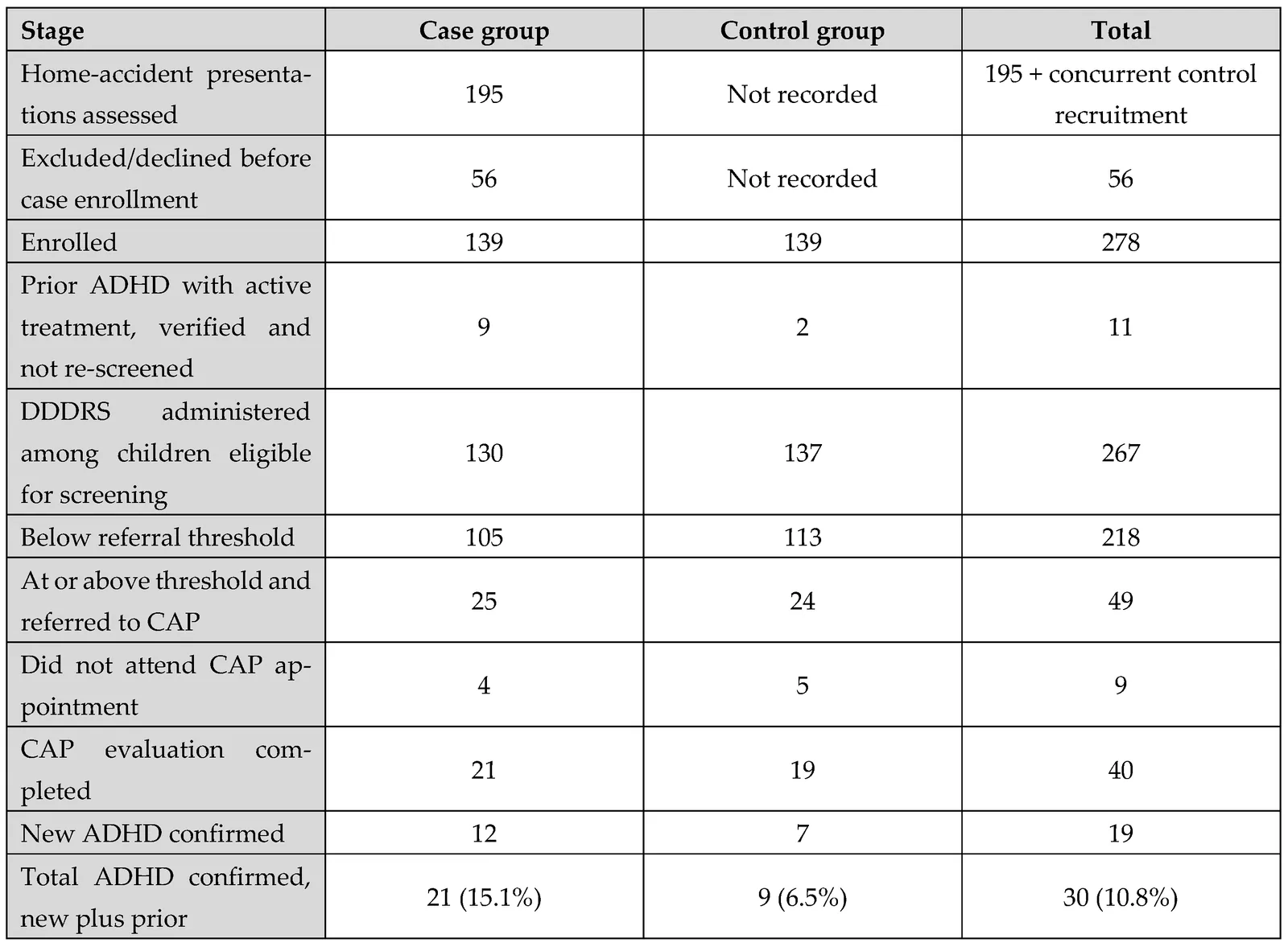
STROBE-oriented participant flow diagram based on available recruitment data. The study dataset did not retain detailed control-candidate screening counts, center-specific recruitment numbers, recruitment-hour logs, or a breakdown of the 56 excluded/declining case presentations; this limitation is stated in the Methods and Limitations. ADHD = attention-deficit/hyperactivity disorder; CAP = Child and Adolescent Psychiatry; DDDRS = Turgay DSM-IV Disruptive Behavior Disorders Rating Scale.

During the enrollment period, 195 children aged 5–11 years presented with a home accident at the two study EDs. Fifty-six children (28.7%) were excluded because they did not meet inclusion criteria or declined participation, leaving 139 in the case group. The available anonymized dataset did not retain a breakdown of the 56 exclusions by individual reason. A concurrent control group of 139 children was enrolled; the number of potentially eligible controls approached, excluded, or declining participation was not retained in the analysis file. The complete participant flow based on available data is shown in Figure 1.

Of the 278 enrolled children, 11 had a prior documented ADHD diagnosis with active treatment (case: n = 9; control: n = 2) and were classified as ADHD-confirmed after record verification without re-screening. The remaining 267 children completed the DDDRS (case: n = 130; control: n = 137), corresponding to 100% completion among children eligible for screening and 96.0% of all enrolled participants. Forty-nine children (18.4%) met the referral threshold (case: n = 25 [19.2%]; control: n = 24 [17.5%]) and were referred to CAP. Forty of these 49 referred children attended the CAP appointment, giving a specialist follow-through rate of 81.6%. Among the 40 who completed CAP evaluation, 12 case-group children (57.1%) and 7 control-group children (36.8%) received a new ADHD diagnosis. Feasibility endpoints are summarized in Table 1.

### 3.2. Baseline Characteristics

Baseline characteristics are presented in Table 2. Sex distributions were similar between groups (case: 47.5% male; control: 49.6% male; *p* = 0.719). Mean age was 8.5 years (SD 1.73) in the case group and 8.2 years (SD 1.81) in controls (*p* = 0.132). Grade distributions were also comparable (*p* = 0.819). Prior ADHD was more common in the case group (6.5% vs. 1.4%), but the difference did not reach statistical significance by Fisher’s exact test (*p* = 0.060).

### 3.3. Secondary Exploratory Outcome: ADHD Diagnosis by Case-Control Status

ADHD was confirmed in 21 of 139 case-group children (15.1%) versus 9 of 139 controls (6.5%) (chi-square *p* = 0.020; Fisher’s exact *p* = 0.032; unadjusted OR, 2.57; 95% CI, 1.13–5.83) (Table 3). In an exploratory multivariable logistic regression model with home-accident case status as the dependent variable and confirmed ADHD as the principal independent variable, adjustment for sex, age, and school grade yielded a similar estimate (adjusted OR, 2.45; 95% CI, 1.06–5.65; *p* = 0.035). Study center could not be included in this model because center-specific individual-level data were not available in the anonymized analysis dataset. Among all ADHD-confirmed children, 12 were newly diagnosed following CAP evaluation in the case group and 7 in the control group; 11 had prior treated ADHD (case: n = 9; control: n = 2).

A complete-case sensitivity analysis excluding children who did not attend CAP evaluation yielded similar results: ADHD was confirmed in 21 of 135 case-group children and 9 of 134 controls (OR, 2.56; 95% CI, 1.13–5.82; chi-square *p* = 0.021; Fisher’s exact *p* = 0.032). Among children without prior treated ADHD, newly diagnosed ADHD was observed in 12 of 130 cases (9.2%) versus 7 of 137 controls (5.1%) (OR, 1.89; 95% CI, 0.72–4.96; *p* = 0.190). This sensitivity analysis was not statistically significant, indicating that the overall case-control difference was partly attributable to the numerical imbalance in pre-existing ADHD between groups and should be interpreted accordingly.

### 3.4. DDDRS Screening Positivity

Among the 267 participants who completed the DDDRS, 49 children (18.4%) met the referral threshold: 25 of 130 case-group participants (19.2%) and 24 of 137 controls (17.5%). This difference was not statistically significant (Pearson’s chi-square *p* = 0.718), confirming that screening positivity was comparable between groups. Because screen-negative children were not referred for CAP evaluation, the study cannot estimate DDDRS sensitivity or specificity in this ED pathway.

### 3.5. Subgroup Findings

Sex distribution is presented descriptively in Table 4. Among the 21 ADHD-confirmed case-group children, 13 were male (19.7% of 66 boys) and 8 were female (11.0% of 73 girls). This numerical difference was not statistically significant (Fisher’s exact *p* = 0.164). In the control group, 4 of 69 boys (5.8%) and 5 of 70 girls (7.1%) were ADHD-confirmed (*p* = 0.745). The near-equal male/female distribution of ADHD-confirmed controls was unexpected compared with typical clinical ADHD sex ratios; however, it was based on only nine control ADHD cases and should be interpreted as random variation in a small selected ED control sample rather than evidence of a true sex pattern.

Age distribution is presented descriptively in Table 5. Within the case group, ADHD was confirmed in 4 of 35 children aged 5–7 years (11.4%), 11 of 63 children aged 8–9 years (17.5%), and 6 of 41 children aged 10–11 years (14.6%). Differences across age bands were not statistically significant (chi-square *p* = 0.723). A sensitivity analysis restricted to children aged 6–11 years excluded three 5-year-old controls and yielded a similar exploratory association between confirmed ADHD and home-accident case status (21/139 [15.1%] vs. 9/136 [6.6%]; unadjusted OR, 2.51; 95% CI, 1.11–5.70; *p* = 0.024; sex-, age-, and grade-adjusted OR, 2.42; 95% CI, 1.05–5.57; *p* = 0.038).

### 3.6. Injury Mechanisms and Day-of-Week Pattern

The most common injury mechanism was falling (n = 76, 54.7% of 139 case-group presentations), followed by blunt trauma or collision (n = 29, 20.9%), burn (n = 14, 10.1%), ingestion of a foreign body or toxic substance (n = 10, 7.2%), sharp-object injury (n = 9, 6.5%), and other domestic injury (n = 1, 0.7%) (Table 6). Home accidents were most frequent on Sundays (n = 32, 23.0% of case-group presentations) and Fridays (n = 26, 18.7%), and least frequent on midweek days.

### 3.7. ED Disposition

Of 278 participants, 254 (91.4%) were discharged after ED management. Twenty-two children (7.9%) required ward admission and two (0.7%) were admitted to the pediatric intensive care unit; both intensive care unit admissions were from the case group. The overall difference in final disposition between groups was not statistically significant (*p* = 0.238). Disposition is reported as a secondary descriptive outcome; the study was not powered to detect differences in clinical severity.

## 4. Discussion

This prospective two-center study primarily demonstrates that an ED-based two-step ADHD screening and specialist referral pathway after pediatric home-accident presentation is operationally feasible. All children eligible for ED screening completed the DDDRS, 18.4% met the referral threshold, and 81.6% of referred children attended CAP evaluation. These findings suggest that the ED encounter can be used pragmatically to identify a subgroup of children for whom specialist ADHD evaluation is clinically reasonable, while recognizing that screening is not equivalent to diagnosis.

The secondary exploratory case-control comparison found that primary school-age children presenting to the ED following a home accident had a higher overall frequency of confirmed ADHD than non-injured controls (15.1% vs. 6.5%; unadjusted OR, 2.57; 95% CI, 1.13–5.83; *p* = 0.020). The exploratory multivariable logistic regression model adjusted for sex, age, and school grade produced a similar estimate (adjusted OR, 2.45; 95% CI, 1.06–5.65; *p* = 0.035). However, this finding requires cautious interpretation because the sensitivity analysis restricted to children without prior treated ADHD was not statistically significant (OR, 1.89; 95% CI, 0.72–4.96; *p* = 0.190). Therefore, the overall association appears partly driven by the numerical imbalance in pre-existing ADHD between groups (9 vs. 2 children with prior treated ADHD; Fisher’s exact *p* = 0.060). Whether this imbalance reflects genuine vulnerability to home accidents among children with known ADHD or chance variation cannot be resolved from this sample.

This revised interpretation is consistent with contemporary evidence while avoiding overstatement. ADHD has been associated with increased accident and injury risk in systematic review evidence across the lifespan [14], and pediatric studies involving head trauma and ear-nose-throat injuries have reported higher ADHD frequencies among injured than non-injured children [15,16]. The mechanistic basis is plausible: ADHD deficits in response inhibition, working memory, and attentional persistence may reduce a child’s ability to anticipate domestic hazards and interrupt impulsive physical actions [12,13]. Recent evidence also links ADHD symptoms with motor development and functional performance in school-aged children, further supporting the relevance of neurodevelopmental vulnerabilities to daily safety behaviors [21].

The predominance of falls (54.7%) as the injury mechanism is consistent with recent and historical pediatric home-injury literature, implicating height-related domestic hazards as principal modifiable risks [7,8,22,23]. The clustering of accidents on Sundays and Fridays suggests that weekend time, changes in supervision, and cumulative fatigue may contribute to injury opportunity, consistent with previous work on weekly variation in childhood injuries [24]. In children with attentional difficulties and impulsivity, this combination of opportunity and reduced hazard anticipation may be particularly relevant.

The diagnostic pathway deserves careful interpretation. The DDDRS was used as a referral screen and not as a diagnostic tool. Final confirmation required CAP assessment or verification of documented prior ADHD [25,26]. Contemporary diagnostic literature emphasizes that rating scales can support identification but should be interpreted alongside clinical assessment and information from multiple settings where possible [6,20]. In the present ED-based pathway, teacher ratings were not available, and screen-negative children were not evaluated by CAP; therefore, the true prevalence of ADHD may have been underestimated and diagnostic sensitivity could not be calculated.

Medication is another important unresolved issue. Children with prior treated ADHD were counted as confirmed after record verification because active treatment may reduce observable symptoms and produce false-negative parent ratings. At the same time, treatment is intended to reduce inattention and impulsivity and may therefore modify injury risk. Because medication type, dose, treatment duration, and adherence were not systematically collected, this study cannot determine whether treated ADHD was associated with lower, higher, or unchanged home-accident risk. Future studies should collect medication exposure and adherence prospectively.

Comorbidity also limits interpretation. Children with psychiatric disorders other than ADHD were excluded to reduce heterogeneity, but ADHD commonly co-occurs with oppositional defiant disorder, conduct problems, anxiety disorders, specific phobias, enuresis, and learning/neurodevelopmental disorders [17,18]. Therefore, this study estimates the feasibility of an ED pathway in a selected sample and should not be interpreted as representing all children with ADHD seen in routine clinical practice.

### Strengths and Limitations

Strengths include the prospective two-center design, use of a validated Turkish screening instrument, specialist CAP confirmation for screen-positive children, blinding of CAP specialists to group allocation, transparent sensitivity analyses, and explicit reframing of the case-control comparison as secondary and exploratory. The groups were comparable on sex and age. To our knowledge, this is the first study to evaluate a two-step ED ADHD screening/referral pathway in primary school-age children presenting with home accidents.

Several limitations should be acknowledged. First, the original sample size calculation was insufficient for a definitive etiologic comparison: re-evaluation indicates that approximately 267 participants per group would have been needed to detect a 13% vs. 6% difference with 80% power, whereas the actual sample provided approximately 53% power. Second, recruitment logs were incomplete: the number of potential control participants assessed, excluded, or declining participation; the detailed reasons for the 56 case exclusions; recruitment hours and days; and center-specific recruitment counts were not retained. Third, only screen-positive children and those with prior documented ADHD underwent specialist evaluation; screen-negative status remained unverified, so the sensitivity and specificity of the DDDRS referral threshold in this ED setting cannot be estimated. Fourth, the diagnostic assessment was a specialist clinical DSM-5 evaluation rather than a structured research interview, teacher forms were not obtained, inter-rater reliability was not calculated, DSM-5 severity specifiers and DDDRS symptom counts were not systematically recorded, and formal intellectual testing was not performed. Fifth, prior treated ADHD cases were verified but medication type, dose, duration, and adherence were not systematically captured. Sixth, the sample excluded comorbid psychiatric disorders, which limits external validity to the broader ADHD population. Seventh, the study excluded traffic, school, sport, and public-space injuries; because ADHD may increase injury risk across settings, the present findings should be interpreted as home-accident-specific feasibility data rather than evidence that ADHD independently increases all injury risk. Eighth, although an exploratory multivariable logistic regression model adjusted for sex, age, and school grade was added during revision, residual confounding remains possible because study center, parental ADHD, socioeconomic position, household crowding, housing hazards, supervision, previous injuries, medication exposure, and comorbid neurodevelopmental conditions were not available as individual-level covariates.

## 5. Conclusions

In this prospective two-center sample, a two-step ED pathway using brief DDDRS screening followed by Child and Adolescent Psychiatry referral was feasible for primary school-age children presenting with home accidents. The case-control comparison identified a higher overall frequency of confirmed ADHD among home-accident cases than among non-injured ED controls, including after exploratory adjustment for sex, age, and school grade. However, this finding should be considered secondary and exploratory because it was partly influenced by imbalance in pre-existing ADHD and was not statistically significant when restricted to newly diagnosed ADHD. The study supports the practicality of ED-based referral screening but does not establish ADHD as an independent cause of home accidents or confirms that screening reduces future injuries. Larger, adequately powered, prospectively adjusted studies with center-level data, structured diagnostic assessment, medication data, comorbidity characterization, and longitudinal injury follow-up are needed.

## Figures and Tables

**Table 1:** Feasibility outcomes of the two-step ED ADHD screening and referral pathway.

Feasibility Endpoint	n/N	Percentage
DDDRS completion among children eligible for ED screening	267/267	100%
DDDRS completion among all enrolled children	267/278	96.0%
Referral-threshold positivity among screened children	49/267	18.4%
CAP attendance among referred screen-positive children	40/49	81.6%
CAP non-attendance among referred screen-positive children	9/49	18.4%
New ADHD confirmation among CAP-evaluated children	19/40	47.5%

ADHD = attention-deficit/hyperactivity disorder; CAP = Child and Adolescent Psychiatry; DDDRS = Turgay DSM-IV Disruptive Behavior Disorders Rating Scale; ED = emergency department.

**Table 2:** Baseline characteristics of the case and control groups.

Characteristic	Case (n = 139)	Control (n = 139)	*p*-Value
Male sex, n (%)	66 (47.5%)	69 (49.6%)	0.719 a
Female sex, n (%)	73 (52.5%)	70 (50.4%)	
Mean age, years (SD)	8.5 (1.73)	8.2 (1.81)	0.132 b
Age range, years	5–11	5–11	
Prior ADHD with active treatment, n (%)	9 (6.5%)	2 (1.4%)	0.060 c
Grade distribution (1/2/3/4)	19/38/42/40	25/30/41/43	0.819 a
Most common ED reason in controls	-	URTI (15.1%); AGE (13.7%); abdominal pain (10.1%)	

a Pearson’s chi-square test; b independent-samples *t*-test; c Fisher’s exact test. ADHD = attention-deficit/hyperactivity disorder; AGE = acute gastroenteritis; ED = emergency department; SD = standard deviation; URTI = upper respiratory tract infection.

**Table 3:** ADHD diagnosis in the case and control groups.

Analysis	Case	Control	*p*-Value	OR	95% CI
Total ADHD, secondary exploratory outcome	21/139 (15.1%)	9/139 (6.5%)	0.020 a	2.57	1.13–5.83
Multivariable logistic regression	-	-	0.035 c	2.45	1.06–5.65
Prior ADHD with active treatment	9/139 (6.5%)	2/139 (1.4%)	0.060 b	-	-
Newly diagnosed ADHD among children without prior ADHD	12/130 (9.2%)	7/137 (5.1%)	0.190 a	1.89	0.72–4.96
Complete-case sensitivity analysis	21/135 (15.6%)	9/134 (6.7%)	0.021 a	2.56	1.13–5.82
Sensitivity analysis restricted to age 6–11 years	21/139 (15.1%)	9/136 (6.6%)	0.024 d	2.51	1.11–5.70

a Pearson’s chi-square test; b Fisher’s exact test; c logistic regression adjusted for sex, age, and school grade; d sensitivity analysis restricted to children aged 6–11 years. Non-attenders to CAP evaluation (case n = 4; control n = 5) were classified as ADHD-unconfirmed in the primary analysis. ADHD = attention-deficit/hyperactivity disorder; CI = confidence interval; OR = odds ratio.

**Table 4:** Sex distribution of ADHD-confirmed children by group.

Group	Sex (n)	ADHD Confirmed, n (%)	ADHD Not Confirmed, n (%)	Fisher *p*
Case (n = 139)	Male (n = 66)	13 (19.7%)	53 (80.3%)	0.164
Case (n = 139)	Female (n = 73)	8 (11.0%)	65 (89.0%)	
Control (n = 139)	Male (n = 69)	4 (5.8%)	65 (94.2%)	0.745
Control (n = 139)	Female (n = 70)	5 (7.1%)	65 (92.9%)	

Fisher’s exact test compared ADHD frequency between sexes within each group. Percentages are row percentages, with the denominator equal to the total number in each sex subgroup. ADHD = attention-deficit/hyperactivity disorder.

**Table 5:** ADHD diagnosis by age group in the case group.

Age Group	n in Band	ADHD Confirmed, n (%)	ADHD Not Confirmed, n (%)	Chi-Square *p*
5–7 years	35	4 (11.4%)	31 (88.6%)	
8–9 years	63	11 (17.5%)	52 (82.5%)	0.723
10–11 years	41	6 (14.6%)	35 (85.4%)	
Total	139	21 (15.1%)	118 (84.9%)	

Pearson’s chi-square test was used across three age bands. The result was non-significant; data are descriptive. ADHD = attention-deficit/hyperactivity disorder.

**Table 6:** Mechanisms of home accidents in the case group (n = 139).

Mechanism	n (% of 139)
Falling	76 (54.7%)
Blunt trauma or collision	29 (20.9%)
Burn	14 (10.1%)
Ingestion of foreign body or toxic substance	10 (7.2%)
Sharp-object injury	9 (6.5%)
Other domestic injury	1 (0.7%)

## Data Availability

The data presented in this study are available on reasonable request from the corresponding author. The data are not publicly available because of privacy and ethical restrictions involving pediatric participant data.

## Abbreviations

ADHD, attention-deficit/hyperactivity disorder; CAP, Child and Adolescent Psychiatry; CI, confidence interval; DDDRS, Turgay DSM-IV Disruptive Behavior Disorders Rating Scale; ED, emergency department; OR, odds ratio.
